# Care Integration for Hepatitis C Virus Treatment Through Facilitated Telemedicine Within Opioid Treatment Programs: Qualitative Study

**DOI:** 10.2196/53049

**Published:** 2024-06-12

**Authors:** Suzanne S Dickerson, Saliyah J George, Ana Ventuneac, Arpan Dharia, Andrew H Talal

**Affiliations:** 1 Division of Tenured and Tenure-track Faculty Development, School of Nursing University at Buffalo State University of New York Buffalo, NY United States; 2 Division of Liver Diseases Department of Medicine Icahn School of Medicine at Mount Sinai New York, NY United States; 3 START Treatment & Recovery Centers Brooklyn, NY United States; 4 Division of Gastroenterology, Hepatology and Nutrition Jacobs School of Medicine and Biomedical Sciences University at Buffalo, State University of New York Buffalo, NY United States

**Keywords:** hepatitis C virus, integrated treatment, facilitated telemedicine, substance users, people with opioid use disorder, opioid, opioids, telemedicine, telehealth, eHealth, e-health, ICT, substance use, substance abuse, HCV, hepatitis, liver, interview, interviews, qualitative, hermeneutic, phenomenological, implementation, integration, experience, experiences, attitude, attitudes, opinion, perception, perceptions, perspective, perspectives, addict, addiction, addictions, addicts, hepatic

## Abstract

**Background:**

Telemedicine has the potential to remove geographic and temporal obstacles to health care access. Whether and how telemedicine can increase health care access for underserved populations remains an open question. To address this issue, we integrated facilitated telemedicine encounters for the management of hepatitis C virus (HCV), a highly prevalent condition among people with opioid use disorder (OUD), into opioid treatment programs (OTPs). In New York State, OTPs are methadone-dispensing centers that provide patient-centered, evidence-based treatment for OUD. We investigated the integration and impact of facilitated telemedicine into OTP workflows in these settings.

**Objective:**

This study aims to understand OTP staff experiences with integrating facilitated telemedicine for HCV treatment into OTPs, including best practices and lessons learned.

**Methods:**

We conducted semistructured interviews with 45 OTP staff members (13 clinical, 12 administrative, 6 physicians, and 14 support staff members) at least one year after the implementation of facilitated telemedicine for HCV management. We used hermeneutic phenomenological analysis to understand OTP staff experiences.

**Results:**

We identified 4 overarching themes illustrating the successful integration of facilitated telemedicine for HCV care into OTPs. First, integration requires an understanding of the challenges, goals, and values of the OTP. As OTP staff learned about new, highly effective HCV therapies, they valued an HCV cure as a “win” for their patients and were excited about the potential to eliminate a highly prevalent infectious disease. Second, the integration of facilitated telemedicine into OTPs fosters social support and reinforces relationships between patients and OTP staff. OTP staff appreciated the ability to have “eyes on” patients during telemedicine encounters to assess body language, a necessary component of OUD management. Third, participants described high levels of interprofessional collaboration as a care team that included the blurring of lines between disciplines working toward a common goal of improving patient care. Study case managers were integrated into OTP workflows and established communication channels to improve patient outcomes. Fourth, administrators endorsed the sustained and future expansion of facilitated telemedicine to address comorbidities.

**Conclusions:**

OTP staff were highly enthusiastic about facilitated telemedicine for an underserved population. They described high levels of collaboration and integration comparable to relevant integrative frameworks. When situated within OTPs, facilitated telemedicine is a high-value application of telemedicine that provides support for underserved populations necessary for high-quality health care. These experiences support sustaining and scaling facilitated telemedicine in comparable settings and evaluating its ability to address other comorbidities.

**Trial Registration:**

ClinicalTrials.gov NCT02933970; https://clinicaltrials.gov/study/NCT02933970

## Introduction

Care integration adds value to existing health care venues by providing new point-of-care options, especially for underserved populations. Fragmented health care is a prevailing problem, exemplified by the separation of medical and behavioral health care [1]. The artificial separation can be particularly problematic for stigmatized populations, such as people with opioid use disorder (OUD), who frequently face difficulties navigating and accessing traditional health care settings [2]. Additionally, people with OUD have high hepatitis C virus (HCV) incidence and prevalence due to injection drug use and limited access to HCV therapy [3]. To achieve HCV elimination by 2030, as proposed in several jurisdictions [4-6], HCV treatment access must increase.

We sought to address restricted HCV care access for people with OUD through a randomized controlled trial of care integration using technology that is at the intersection of the internet or videoconferencing, and health care. Specifically, we used a facilitated telemedicine model integrated into 12 opioid treatment programs (OTPs) throughout New York State [7,8]. Facilitated telemedicine is an approach to health care delivery that combines social interactions between patients and staff (ie, case managers and clinical staff) with bidirectional videoconferencing between patients and geographically separated providers to promote trust. Facilitated telemedicine can integrate HCV treatment into sites, such as OTPs, that are convenient and familiar to patients with OUD but that lack HCV services and infrastructure. Methadone dispensing in the United States is highly structured and requires attendance at a licensed OTP, a system that was created through specific regulations [9]. A physician or advanced practice provider initially evaluates patients, and nurses administer methadone under direct observation.

OTPs in New York State assess, titrate, and dispense methadone in combination with patient-centered counseling to manage people with OUD, including symptoms of opioid withdrawal [2,10,11]. These approaches, combined with onsite medical services such as phlebotomy and HCV medication dispensing in an OTP setting, promote the integration of HCV treatment into OUD care. While OTPs can be stigmatizing, in our experience, people with OUD generally consider OTPs destigmatizing; they can be viewed as “safe spaces” and potential “medical homes” for integrating expanded health care delivery [12,13]. We integrated facilitated telemedicine into OTPs to connect patients with HCV specialists.

Since knowledge is limited concerning staff considerations that affect facilitated telemedicine integration into OTP settings, we investigated OTP staff’s experiences of facilitated telemedicine integration for HCV care into OTPs. We used the sociotechnical system framework to identify telemedicine delivery issues and potential sociotechnical concerns for people with OUD [14]. We sought to understand the meaning of facilitated telemedicine integration for staff in everyday clinical practice and the role that staff played in the facilitation. Our goal is to provide insights and potential considerations for future integration. Investigating staff’s common meanings and shared practices promotes understanding of integration successes, challenges, and lessons learned. We compared our results to the conceptual care integration framework developed by the Center for Integrated Health Solutions [15].

## Methods

### Parent Study Summary

We recently completed a stepped-wedge, randomized controlled trial comparing HCV treatment through facilitated telemedicine integrated into 12 OTPs throughout New York State compared to offsite referral (usual care) [16]. In New York State, OTPs are methadone-dispensing centers that provide patient-centered harm reduction approaches using an evidence-based medical treatment for OUD [10]. Although methadone as an OUD treatment is supported by New York State Medicaid, OTPs do not routinely offer primary care. Of the 12 study sites, 4 were community organizations, 4 were university-affiliated, and 4 were affiliated with health systems. In total, 10 of the 12 sites had their own institutional review boards; the board from the parent study site covered the other 2 sites. At the initiation of the study (ie, 2017), 10 of 12 sites offered onsite phlebotomy. By the end of the study (ie, 2022), all sites offered onsite phlebotomy. We commenced participant recruitment on March 1, 2017, and concluded on February 29, 2020.

We recruited a total of 602 participants. Both in-person and telemedicine encounters were managed by hepatitis specialists, who forwarded notes of the HCV consultation to the OTP for inclusion in the medical record. The entire facilitated telemedicine encounter was integrated into the OTP with bidirectional videoconferencing between the patient-participant and the hepatitis provider. Third-party coverage of clinical care and direct-acting antivirals were study inclusion criteria. A study-supported case manager was the research team representative at each study site and was credentialed in human subjects training. The case manager facilitated the telemedicine encounter and was also an HCV educator, patient navigator, and advocate. Direct-acting antivirals for HCV treatment were dispensed in the OTP alongside methadone for OUD. Take-home doses were available on a case-by-case basis. Subsequently, we followed cured participants for 2 years posttreatment cessation to assess for reinfection.

### Methodology

We used hermeneutic phenomenology to reveal understandings of human situations as experienced within a context of time, place, and situational influences [17,18]. In this approach, we gain an understanding of the experience of a technology from the perspective of health care as a human experience interacting with one another [19] versus a replacement of social aspects of care. In a postpositivist or interpretive framework, hermeneutic phenomenology uses an a priori (ie, no preconceived theory) approach to formulate open-ended questions, interpret the interview text, and explicate common meanings and shared practices. Themes are revealed through the interpretation of the language of experiences, as portrayed in staff narrative interviews. For details concerning hermeneutic phenomenology, please see [20,21] and Multimedia Appendices 1 and 2 [20,22-27].

### Setting and Recruitment

We used purposive sampling to recruit OTP staff and administrators involved in HCV care integration at least one year after the initiation of facilitated telemedicine. We interviewed approximately 4 participants per site, representing 11 sites. We interviewed 45 participants, including 16 clinical, 15 administrative, and 14 support staff members (Table 1).

**Table 1:** Demographics of the 45 participants interviewed.

Characteristic			Participants, n (%)
**Age (years)**			
	21-40	9 (20)	
	41-60	26 (58)	
	61-80	9 (20)	
	Not disclosed	1 (2)	
**Sex**			
	Male	17 (38)	
	Female	28 (62)	
**Race**			
	Black	18 (40)	
	White	22 (49)	
	Other (Latino, Puerto Rican, or mixed)	3 (7)	
	Not disclosed	2 (4)	
**Ethnicity**			
	Hispanic	3 (7)	
	Non-Hispanic	38 (84)	
	Other (this included Jamaican and Jewish)	2 (4)	
	Not disclosed	2 (4)	
**Highest educational attainment**			
	Less than bachelor’s	4 (9)	
	Bachelor’s	13 (29)	
	More than bachelor’s	11 (24)	
	Professional certification-board certified, LMSW^a^, PA^b^, or CASAC^c^	17 (38)	
**Staff type**			
	Administrator (directors or program administrators)	12 (27)	
	Clinical (registered nurse, nurse practitioner, or PA)	13 (29)	
	Physicians	6 (13)	
	Support (counselors, patient account rep, or others)	14 (31)	

^a^LMSW: licensed medical social worker.

^b^PA: physician assistant.

^c^CASAC: certified alcohol and substance abuse counselor.

### Interview Conduct

SSD interviewed all participants from January to July 2021. We developed separate interview guides for staff and administrators (Multimedia Appendix 2). The initial open-ended question inquired about what the participants viewed as the most important aspect of integrated care through facilitated telemedicine. If not discussed in the open-ended inquiry, we subsequently used probes for further elaboration on participants’ experiences of facilitated telemedicine and integration frameworks [14,21]. Interviews were recorded by Zoom (Zoom Video Communications), transcribed, deidentified, and verified by staff by comparing transcriptions against the recording for accuracy. One participant opted for an audio-only recording. The interviews lasted 30-60 minutes, and no participant prematurely discontinued the interview. The interviewer confirmed the final transcript version before the analysis.

### Analysis

The multidisciplinary analysis team included a hermeneutic phenomenology expert (SSD), a social psychologist (AV), and a case manager (SJG), who performed the initial thematic coding. Subsequently, the study principal investigator (AHT) and study director (AD) joined the analysis team for the final coalescence of themes. SSD guided the analysis using de Witt and Ploeg’s [22] framework for rigor while simultaneously maintaining balanced integration between the participants’ voices and the researchers’ interpretations. The analysis team interpreted the transcripts and field notes in a reflective process that followed iterative steps [20,23]. The team met weekly to identify emergent themes with supportive quotes, which were discussed and refined in an iterative fashion. The team achieved openness by exposing the team’s preunderstandings (biases) through a continuous, systematic process of auditing the team’s interpretations to support theme development consistent with the participants’ experiences. Any thematic disagreements among team members resulted in a return to the original interview transcript for verification. By team consensus, new themes were added as relevant, and the process continued until themes were refined to explicate the phenomenon. The final themes were examined by team members for coherence and comprehensiveness, providing a warranted interpretation.

### Ethical Considerations

The study was approved by the institutional review board at the University of Buffalo (parent institution; STUDY00000724) and by the institutional review boards at each of the sites where the study was conducted. All participants signed informed consent and provided verbal consent reconfirmation before the interview. No participants opted out of the study. All data transcripts were deidentified. Participants received US $25 for their participation, if permissible. Included in this article is an aggregate summary of the data generated during the study. Confidentiality concerns prohibit us from sharing individual data transcripts.

## Results

**Figure 1:**
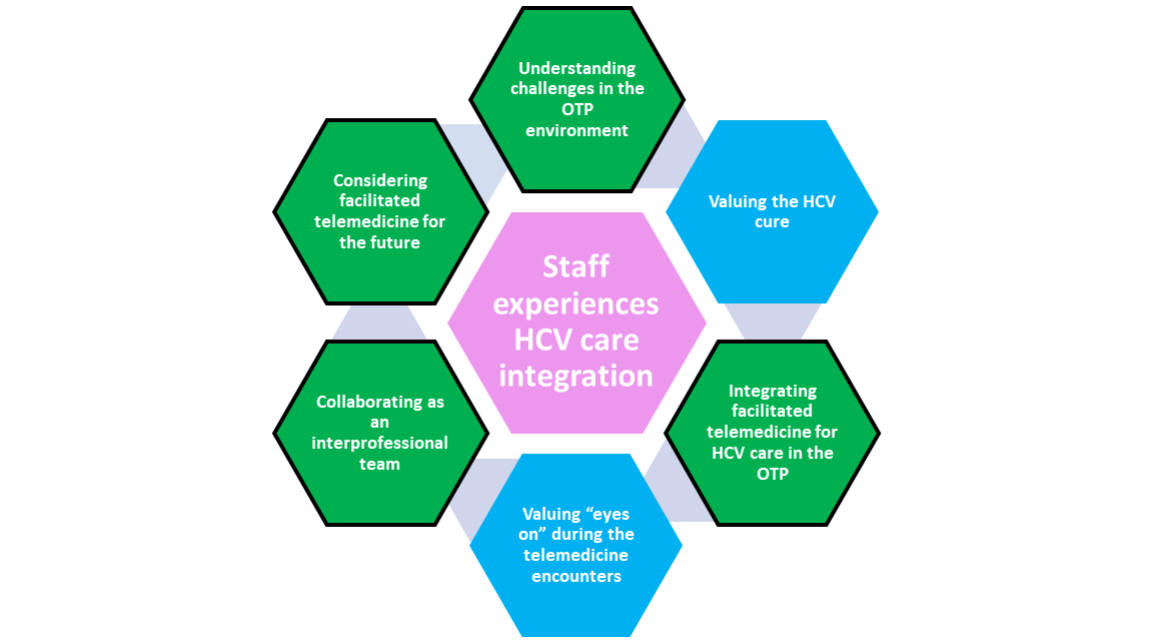
Staff experiences of hepatitis C virus (HCV) care integration are illustrated as themes (green) and subthemes (blue). OTP: opioid treatment program.

Participant interviews revealed 4 themes related to the integration of facilitated telemedicine into a behavioral health setting (Figure 1). OTP staff and administrators shared their experiences of HCV care through facilitated telemedicine integrated into OTPs.

### Theme 1: Understanding Challenges in the OTP Environment as a Point of HCV Care for People With OUD

#### Overview

In the context of working in the OTP, participants reflected on their common experiences managing physical withdrawal symptoms and the psychological effects of the OUD. Staff frequently addressed barriers to care, such as the patients’ competing priorities as well as the difficulties engaging people with OUD who have a “disease of the brain.” Staff narratives additionally explained situations when patients previously attempted to access health care outside of the OTP that were frequently accompanied by unjustified scorn. One staff member explained:

If [patients] go to their primary doctor and say, “I’m addicted to opiates, I’m on methadone,” some doctors won’t even treat them, they will shun them.

Participants alternatively felt that people with OUD considered the OTP environment a safe, destigmatizing space in which to receive health care. Thus, the OTP was an appropriate place in which to provide point-of-care treatment for HCV. As one staff member reflected:

Care integration makes so much sense, we should have done it long ago.... Here is a subset of a population you are not going to reach unless you’re willing to do something like this.

OTP staff create nonjudgmental environments where patients feel comfortable and accepted to build patient trust. As one administrator noted, “[Patients] understand they’re here for treatment, they’re surrounded by people who care about them, who want to listen.” Another administrator reflected that OTP staff adopted a problem-solving, patient-centered approach to OUD treatment by, “trying to meet, treat patients in an individualized way.” Another participant reflected on the care that OTP staff seek to provide: “We’re not here to judge them, we’re here to take care of them.” Staff also face challenges in hectic OTP environments that have large patient loads, as one administrator reflected:

The staff overall do a remarkable job under challenging circumstances with inadequate resources, difficult environments [including] challenging [external] communities that don’t want these programs [due to] stigma.

Nonetheless, participants understood that the OTP’s culture fosters their recognition as “safe spaces” for people with OUD. Participants related that successful integration required an understanding of the OTP’s challenges and values.

#### Valuing the HCV Cure

Participants were very excited to learn about offering the benefit of a newer treatment that provides an HCV cure to their patients. Early in the study, however, participants were uncertain if patients would accept the newer treatment option for HCV care. A staff member related, “You may not get patient buy‐in [for HCV treatment] right at the beginning.... They’re in crisis, they could be in withdrawal.” To promote buy-in for HCV treatment, study personnel provided HCV education to promote program endorsement for both staff and patients, as both groups were more familiar with older, less-effective therapeutic regimens. As a staff participant explained, “I was really excited hearing that there were [HCV] medications.... Highly effective.”

As time went on, staff-participants also appreciated the opportunity for onsite HCV treatment to benefit their patients. Staff described how the patients who completed treatment shared their endorsement with other peers and became a peer pipeline for the facilitated telemedicine approach, as one staff member reported:

A number of my patients have been cured and their lives have improved.... When folks were introduced to the possibility of being in the study, they didn’t run from it, they actually ran to it.

Participants appreciated the accessibility and convenience of integrating HCV care into venues already frequented by people with OUD. The enthusiasm for the program grew over time as OTP staff became involved in HCV care integration in the OTP. As one administrator suggested:

Everyone benefited, mostly the patients. Not only did we help save lives, but [we] injected back into the program opportunities for learning and feeling that we’re participating in advancing science.

Participants experienced the merits of an HCV cure through patient success stories of accessing treatment that provided hope for the future. As one staff member reflected, “There’s no need to languish, you can get treatment and there is a better tomorrow.” An administrator commented that patients are “absolutely thrilled [with the] opportunity to have a successful medical intervention, it’s life changing.” In addition, the clinical staff reflected on the observed outcomes of treatment. One said, “Their countenance changed, [it was] brighter. Their level of confidence [increased].” Another clinical staff person commented on the outcomes:

The responses were great, in fact their methadone doses decreased, their mental status got better, their overall symptoms got better. Commitment to the [HCV] treatment gave them another responsibility that helped in their recovery process.

### Theme 2: Integrating Facilitated Telemedicine for HCV Care Into the OTP

#### Overview

Participants were overwhelmingly positive toward HCV treatment through facilitated telemedicine integrated into the OTP that fosters social support and relationship building between staff and patients, which is foundational for effective health care delivery. One staff member reflected:

They build trust here because they come here every day. They’re used to our doctors [and] nurses.... They don’t want to go anywhere else to get treated.

Therefore, a quality program that endorses patient-centered approaches is supported within the OTP even when it includes the technology of telemedicine as long as a concurrent focus is on the social aspects of care, which are foundational to the OTP. One administrator explained that telemedicine “allows increased access to treatment that improves quality and monitoring.” Staff described the integration of behavioral and medical health with infectious disease treatment as creating a “one-stop shop of healthcare delivery.”

Facilitated telemedicine is built on a foundation of preexisting trusting relationships between patients and staff [2,21]. Case managers coordinated the telemedicine encounters, and familiar clinical staff were present during the initial encounter. OTP staff’s presence during the initial telemedicine encounter provided the necessary sociotechnical connections that were adapted to include the facilitated component that occurred within the OTP. A staff member described the essential components of the facilitated telemedicine encounter: “What makes it positive is that they have a familiar person onsite who they’ve already seen. That’s the seamless aspect.” Staff were pleased with the patients’ quick and easy adaptation to facilitated telemedicine when the provider on the screen became familiar to the patients, which resulted in the transfer of trust across the screen. As one staff member explained:

The technology was new, and it took one or two visits and then they became very comfortable with [telemedicine] and talked [as] if the person was in the room.

The presence of case managers during the telemedicine encounters created a bridge between the patient and the HCV specialist, rephrasing questions and addressing patient concerns. One staff person explained how the OTP clinical staff also facilitated the interaction for the specialist by providing essential information and performing clinical assessments: “The PA [was] in the room [during the initial telemedicine encounter].... Someone who knew [the patient] and about [his or her] situation.” Furthermore, physician assistants or nurse practitioners used their clinical skills, adding benefit to the facilitated telemedicine encounter as one clinical staff member explained, “For the initial visits, I did their physical [exam].” The benefit of facilitated telemedicine is bidirectional; the telemedicine provider collaborated with the onsite clinician to perform physical examinations as indicated, and the onsite clinician achieved enhanced job satisfaction through the application of new and enhanced clinical assessments to gauge liver health. Participants further labeled the facilitation as a “warm handoff” and “seamless” transition to the telemedicine provider. Over time, the interactions became more efficient, as one staff participant explained: “[Patients] asked appropriate questions [and] felt comfortable, that really helped with the compliance.... [They] got more involved in their own treatment.” Furthermore, for the remote specialist, the support of those who facilitate the encounter provides the social aspect of the sociotechnical interaction. As one staff participant reported, “If someone is there, who is the specialist’s eyes and ears, then it [telemedicine] works.”

#### Valuing “Eyes On” During Telemedicine Encounters

Staff members who participated in the facilitated telemedicine encounters deliberated regarding the need for having an “eyes on” perspective of patients during the encounters to observe nonverbal cues and OUD-related behavioral manifestations. One clinical staff member compared telemedicine to face-to-face encounters to explain the value of being present in the same room, as necessary, for appropriate assessments:

Often when you’re talking to them [patients], you notice things that look a little odd and that will trigger a question. “You have a black eye, did someone hit you?”

The “eyes on” perspective was described by a clinical staff person as an important skill when assessing individuals’ health:

You really want to lay eyes on the problematic patients that are more likely to be impaired.... When they’re seeing the doctor, [patients] can get their act together.

Counselors agreed on the importance of direct visualization to assess for manifestations of OUD: “Sometimes in the world of addiction psychiatry, body language gives a lot of information.” Some staff also commented on the necessity of “eyes on” for other conditions such as rashes, swelling, and inflammation that could benefit from an “eyes on” perspective.

### Theme 3: Collaborating as an Interprofessional Team

**Figure 2:**
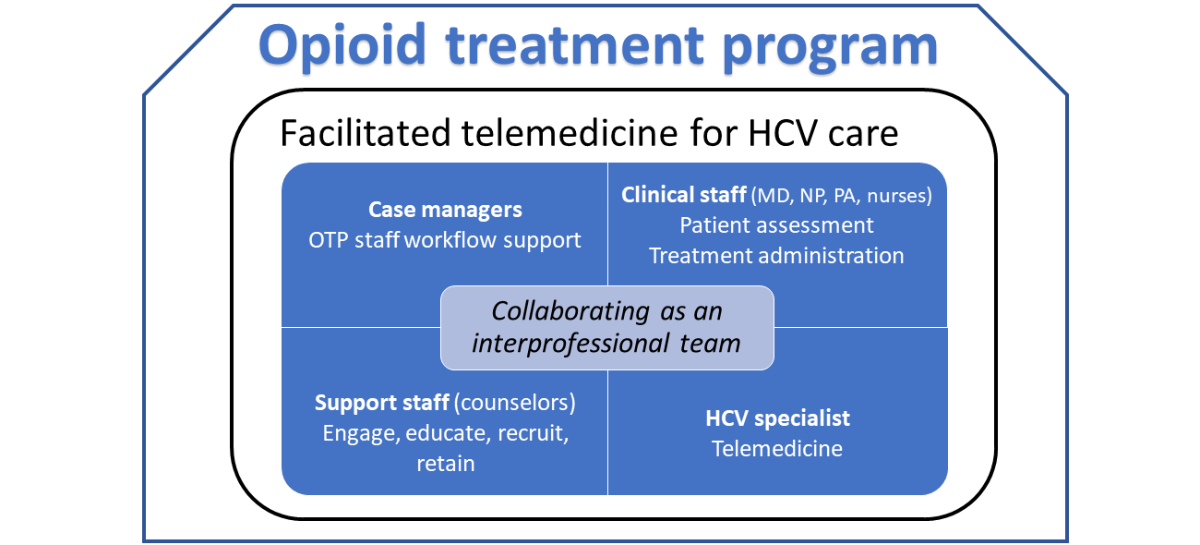
Collaborating as an interprofessional team. Study-supported case managers are integrated into the opioid treatment program (OTP) as critical to supporting OTP staff workflows. Clinical staff, including medical doctors (MDs), nurse practitioners (NPs), and physician assistants (PAs), conduct physical examinations during telemedicine encounters. Nurses coordinate medication storage, dispensing, and take-home doses. Support staff, such as counselors, incorporate hepatitis C virus (HCV) care into the treatment plan through engagement, education, recruitment, and retention of care. The HCV specialist orders medications and labs for HCV management through telemedicine encounters.

The OTP and study staff collaborated as an interprofessional team to integrate facilitated telemedicine into the OTP (Figure 2). This collaboration was initiated and supported by a sentiment of hope and excitement for the ability to offer needed HCV treatment for their patients. Staff spoke of its dedication to supporting an underserved population with a treatment that actually offered a cure for HCV, which was “a big deal.” Staff were excited and open to this opportunity, which some called an innovation. One staff participant concluded:

We’ve learned how to collaborate from the staff and especially the counselors...[who] were excited that their [HCV] patients will be treated. The collaboration was at the highest level possible.

The staff developed daily workflow processes to promote integration within each OTP according to the constellation of staff at each setting. An administrator explained how it worked at one OTP: “The counselors would notify the patients [of] an appointment, the account rep [representative] would remind patients, and the nurse practitioner helped to round up patients.” Another site staff member elaborated, “Between the nurses and the counselors, we all work [together], encouraging the clients to participate, making them feel welcome.”

Participants enthusiastically reported how the study case managers integrated within the OTP staff to focus on the necessary treatment processes. Case managers provided the necessary structure for the staff to participate and integrate HCV treatment through facilitated telemedicine within their usual workflow, positively influencing the OTP staff. Thus, OTP leadership viewed case managers as essential to the OTP workflow as one administrator related: “It really felt like the [case managers] were team members, not outside folks coming in to do a project.” Case managers’ collaborations made the HCV treatment seamless. An administrator reported:

Having the case manager was very helpful because there were so many pieces. If that [HCV treatment] had fallen on our staff, that would have really made this challenging...The case manager knew how to navigate our system.

The navigation included identifying positive HCV cases, understanding specific treatment parameters, obtaining insurance approval for HCV medications, being present during all telemedicine encounters, following the patient through the HCV treatment course, and obtaining outcome measurements. One administrator reflected:

The [case manager role] is important because it allows the counselors to focus on the substance use disorder and the social workers to deal with the mental health issues.

The case managers were also essential for patient enrollment support, as staff described: “The case manager was very involved with all these patients; she was able to relate to them within their world.”

Participants worked collaboratively as a “care team” consisting of clinicians, nurses, counselors, and case managers. Clinicians answered patients’ questions, provided support, and encouraged adherence. Nurses coordinated medication storage, dispensing, and take-home doses. One staff participant explained:

The nurses would get things set up. The client was dosed at the window, and they have their take-out [methadone] bottles and their hep C medicine in the same bag, and off they would go, it was seamless.

Counselors, as part of their routine employment duties, communicated with patients regularly, which enabled case managers to effectively educate, recruit, engage, and retain participants. An administrator reported:

[Facilitated telemedicine] has helped the counselors work with patients towards the treatment plan throughout the course of the treatment, [counselors are] able to provide information, communicate with the patient, and help move things along.

### Theme 4: Considering Facilitated Telemedicine for the Future

OTP administrators endorsed sustaining and growing the facilitated telemedicine model. Participants provided meanings and suggestions for facilitated telemedicine integration (Table 2), and they advocated that the OTP environment provides an important foundation. Administrators agreed that facilitated telemedicine improved care access: “It makes so much sense [and] has made me think about the integration of other services, not just co-location, but real integration.” Participants commonly experienced their patients’ lack of follow-through with offsite health care referrals and suggested that facilitated telemedicine be expanded to address comorbidities. Additionally, participants felt the facilitated telemedicine model could be translated to other OTP settings, as one administrator explained:

[Patients] don’t like the idea of shopping around to different places to get care, so the idea is that they can come here to get their methadone, counseling, medical, primary, HIV [care], and diabetic

care

Staff suggested that additional potential expansion opportunities, especially through telemedicine, include “HIV, AIDS” and “internal medicine and chronic disease management including diabetes and hypertension.” Administrators indicated that their future includes leveraging OTP infrastructure to address comorbid health care issues.

Participants reflected on the positive outcomes of facilitated telemedicine for their patients, endorsing hope for their future health. One staff participant said:

I think having that little success, even just completing hep C treatment, is a win for them, it makes them more willing to treat other medical issues.

However, using telemedicine solely without the facilitated portion could lead to health care that lacks the social component that is so necessary to build relationships and quality care. If health care becomes a standardized protocol devoid of human social interaction, there is a risk of dehumanizing health care. As one staff member related, now after the study, there is a change in HCV care as it is protocol-driven. Thus, if HCV is managed solely with technology, treatment effectiveness and satisfaction may be diminished, especially among underserved populations, such as people with OUD. One staff member said, “The hepatitis treatment is protocol driven. It’s very stepwise in process, a standardized workup...You can get a good workflow down” but not at the risk of eliminating human interaction, trust, and support.

**Table 2:** Participant insights of facilitated telemedicine.

Themes and meanings		Advice with supporting quotes
**Theme 1: Understanding challenges in the OTP^a^ environment as a point of care for people with OUD^b^**		
	Challenges of the OTP environment	Be flexible...to work with the patients, be ready for initial resistance. Get ready for a lot of no shows in the beginning. Persistence. [Staff] [What] would have helped [is being] able to do the blood draws onsite. We had to send our patients to the hospital to get their blood work done and even two blocks [distance] are a hurdle. [Administrator]
	Valuing telemedicine in a familiar environment	Make sure that the client is aware of what telemedicine is, so that they are not scared...This population can be standoffish...So explain “it’s to help you and your health conditions”...Communicate that it’s beneficial and convenient.” [Staff] I would recommend [telemedicine] remain in the program...Convenience and accessibility [are key].” [Staff] One patient was private [and] initially apprehensive. When he realized that he didn’t have to go somewhere else meeting strangers [and he could remain] within the environment [and people] that he’s accustomed to, he willingly participated.” [Staff]
	Understanding the HCV^c^ cure	People who were completely cured would never have been cured...They would never go through the whole series of visits. [Staff] Clients speak to [other] clients about treatment and their experiences. [Staff]
**Theme 2: Integrating facilitated telemedicine for HCV care into the OTP**		
	Understanding facilitated telemedicine	By seeing a telemedicine interaction, you see what is required and what is accomplished. You sing a different tune because you can see that you can solve problems. [Staff] Education is the most important part...Let them know that [telemedicine] is HIPAA-compliant. [Staff] Nothing’s going to be shared, it’s confidential, you have to establish patient trust. [Staff] I think telemedicine has been very helpful because of the ease of treatment...I’ve been astonished by the degree of compliance with treatment. The way we were able to successfully collaborate and integrate the HEP C treatment into the medication assisted treatment, to improve that compliance and to improve the quality and outcome of care, it’s been amazing. [Administrator]
	Onsite accessibility	You don’t have to worry about the weather. Patients get nervous; “Am I going to get a ride to the clinic? What if I miss my appointment with my counselor? Are they going to let my probation officer know that I was noncompliant?” This way [telemedicine] takes so much anxiety away for the patient. [Administrator]
	Information technology infrastructure needs	An onsite IT person to assist when things went wrong would be very helpful. [Administrator] If the Wi-Fi went down, we’d be really stuck. [It] would have helped to have technical backup in place. [Staff] Make sure you have good solid equipment because sometimes if the audio was bad, you always have poor connection, and it takes so long to set up. [Staff] [I would] like an integrated [HCV medication] dispensing record.... Our electronic system didn’t support it. [Staff]
Theme 3: Collaborating as an interprofessional team-clinical support		[The case manager] has the ability to...be inviting to patients...He was enabling people. Choosing the [right] staff is important. [Staff] We provided “directly observed therapy,” where we would give them their medicines when they come to the window. Some of these patients had to take their meds home because they don’t come every day. I had to call patients on a number of occasions [to remind them]. Give a patient a beeper or a smartphone…We would set it [smartphone] up to alarm so that they’ll remember to take the medicine. [Staff] Having a nurse available to do vitals, room a patient, and update medications, would have been nice. [Staff]
Theme 4: Considering facilitated telemedicine for the future-future considerations		“We’re able to reach this subset of population that we would not have otherwise been able to reach This is a very viable approach.... I think it shows that there's a role for this” [Staff]. “People who would benefit the most are freestanding clinics and small clinic networks… These independent clinics really stand to gain a lot. You’re providing more services for your people, and you can really market this as a reason to go [to the clinic] as opposed to somewhere else” [Staff]. If you have clients that have received the treatment, clients speaking to clients about treatment that they have already received and their experiences, that is the most impressionable to other people. [Staff]

^a^OTP: opioid treatment program.

^b^OUD: opioid use disorder.

^c^HCV: hepatitis C virus.

## Discussion

### Principal Results

OTP staff and administrators shared their experiences of HCV care through facilitated telemedicine integrated into OTPs. Participants understood that the OTPs’ culture fosters their recognition as “safe spaces” for people with OUD. Participants related that successful integration required an understanding of the OTP’s challenges and values (theme 1). Participants appreciated the accessibility and convenience of integrating HCV care into venues already frequented by people with OUD. The OTP environment, coupled with the trust and familiarity of individuals conducting telemedicine encounters, potentiates positive provider-patient relationships across the screen [12,13]. Similarly, the literature has identified these factors as essential components of the provider-patient relationship [28]. Participants valued an HCV cure as a “win” for their patients and as a motivator to address life challenges. As a result, participants became committed to HCV cure within their OTPs. The opportunities to interact with hepatitis specialists through facilitated telemedicine improved the provision of specialty care offered to OTP patients (theme 2). Counselors, nurses, and clinicians supported facilitated telemedicine for HCV care, which supplemented internal workflows and enriched job satisfaction. Counselors included HCV management in their patients’ treatment plans, while nurses and clinicians monitored and educated patients. Case managers were responsible for HCV care delivery and were fully integrated into the OTP workflow (theme 3). Similarly, others have recently illustrated how integrated health care offers opportunities to promote interprofessional collaboration [1,29]. Displaying empathy, especially during telemedicine encounters, promotes high patient satisfaction [11,30] and is an essential component of the sociotechnical approach.

### Comparisons With Previous Work and Standardized Frameworks

We compared participants’ experiences to the Center for Integrated Health Solutions framework categories of collaboration, clinical delivery, and patient experience [15]. Participants indicated that they were mission-driven and patient-centered. Related to *collaboration,* participants integrated study-supported case managers into the OTP, established seamless workflows, and blended roles and responsibilities. The COVID-19 pandemic-associated implementation of telemedicine encounters induced similar changes in staff roles, workflows, and patient interactions [31]. Furthermore, amelioration of cultural differences between health care disciplines (ie, differences between medical and behavioral providers, such as interprofessional hierarchies, differences in treatment vocabularies, and variations in staff supervision) has been shown to occur most effectively by developing, supporting, sustaining, and growing integrated care teams [1]. A recent study that used video-observed therapy for HCV was permissive for support service offerings with the involvement of multidisciplinary teams [32]. A previous meta-analysis illustrated improved HCV therapeutic outcomes with the involvement of a multidisciplinary team [33]. Related to clinical delivery, participants described HCV treatment as protocolized, and they successfully adapted site-level OTP workflows to promote facilitated telemedicine integration with social support existent in the OTP [7]. Related to patient experience, facilitated telemedicine expanded access to patient-centered and equitable HCV care. Consistent with the sociotechnical system framework, participants reported that a dedicated, familiar individual (ie, case manager) is critical for successful telemedicine integration [14]. Thus, participants perceived that facilitated telemedicine increased health care quality and value [34,35]. The investigation described here begins to address knowledge gaps, including understanding of telemedicine’s role as a component of behavioral-medical care integration [36].

### Participant Meanings and Practical Advice

Participants recommended shared practices of facilitated telemedicine in the OTP, such as having “eyes on,” that is, direct patient visualization, to assess OUD manifestations [37]. These observations underscore the potential ineffectiveness of audio-only solutions when evaluating people with OUD. Participants indicated that codispensing HCV medications and methadone increases HCV medication adherence and adds value by potentiating improvements in OUD recovery [38]. New York State regulations and OTP administrators permitted the codispensing of HCV medications and methadone. Additionally, managing HCV and OUD has been shown to improve treatment outcomes, adherence, and retention in care [39-41]. Patients’ commitment to achieving an HCV cure improved their confidence and sense of self-worth, including an improved interest in their whole health and overall well-being [2]. Our observations, consistent with others, illustrate that telemedicine interventions for HCV treatment promote retention in OUD care. Treloar et al [42] found that an HCV and OUD colocated treatment model was overwhelmingly successful, largely because of the familiarity between patients and staff in the OTP setting. Other recent data illustrated improved HCV treatment uptake with integrated care [43]. Participants also offered suggestions for future facilitated telemedicine use (theme 4), suggestions supported by previous data illustrating behavioral health integration into different practice settings [1,29] and successful management of primary care conditions through telemedicine (Table 2) [36,44,45].

### Strengths and Limitations

Due to the novelty of facilitated telemedicine targeted at people with OUD, we used hermeneutic phenomenology to investigate OTP staff’s experiences. Examining the technological implications in a protocol-driven world illuminates the value of facilitated telemedicine, which emphasizes the importance of a trusting relationship between patients and providers. This therapeutic alliance creates a bridge to effective use of technology that does not abandon person-centeredness or widen the digital divide. Another study of Indigenous Australian individuals illustrated that telehealth enabled specialist consultations to occur in the safe environment of the Aboriginal Community Controlled Health Services [46]. In that model, an Indigenous health worker facilitated telehealth interactions and communication between community members and specialists. Subsequent investigations should evaluate facilitated telemedicine integration into different settings through implementation frameworks.

In our case, we interviewed participants at least one year after telemedicine initiation and during COVID-19–associated lockdowns, thereby permitting evaluation of longer-term effects, including assessment of site-level, pandemic-associated changes in health care delivery. Our analysis team, which consisted of experts with diverse backgrounds in qualitative analysis, clinical research, and OUD, maintained data credibility [20,22,23]. Our telemedicine encounters were conducted as clinical research, which may limit their transferability to clinical care. Additionally, the OTP sample reflects the environment, policies, and regulations of New York State, which may limit transferability to other states that have less generous substance use treatment policies. In contrast to other states without governmental support for methadone, New York State Medicaid supports methadone as an OUD-indicated therapy. While we acknowledge that OTPs can have substantial limitations in terms of staff and resources, they are generally less stigmatizing than traditional health care settings, according to people with OUD [2,12,13]. Methadone program staff, however, can be viewed as highly mistrustful, which may interfere with the successful integration of HCV therapy [47]. To build trust between patients and OTP staff, promotion of success stories through a peer pipeline is recommended [47,48]. Some OTP regulations may actually hamper access to methadone and may promote patient stigmatization [49]. As a potential approach to decreasing stigma and improving health outcomes, annual staff training on OUD and OUD-related infectious diseases is recommended. In fact, New York State has annual continuing education requirements for OTP staff and providers. While we did not specifically evaluate reimbursement for services delivered through facilitated telemedicine, administrator interviews provided important insights into the financial aspects of facilitated telemedicine [50]. Administrators also commented on how the expansion of telemedicine during the COVID-19 pandemic enhanced reimbursement for telemedicine services performed at OTPs.

### Conclusions

Participants were highly enthusiastic about facilitated telemedicine for HCV care integrated into OTPs. They identified several practices to promote integrated care, including seamless workflows, high-level collaboration, and blurring of lines between disciplines that portended successful HCV care integration. Participants noted the importance of facilitated telemedicine by familiar staff and identified practices to improve its quality and value. Thus, facilitated telemedicine is a high-value application of telemedicine that bridges treatment protocols to human interactions and provides supports that are essential for quality care [51]. The staff participants perceived profound HCV cure-related changes that motivated subsequent improvements in overall health and well-being. Thus, facilitated telemedicine could be promoted to expand access, improve quality, and add value to health care delivery to underserved populations, including people with OUD.

## Appendix

Multimedia Appendix 1 Consolidated Criteria for Reporting Qualitative Studies (COREQ checklist).

Multimedia Appendix 2 Qualitative methodology supplement and interview guides for staff and administrators.

## Abbreviations

HCV: hepatitis C virus
OTP: opioid treatment program
OUD: opioid use disorder

## Data Availability

The data transcripts generated and analyzed during the study are not publicly available due to confidentiality concerns but are available from the corresponding author on reasonable request.
