# Supplementary material for: Care Integration for Hepatitis C Virus Treatment Through Facilitated Telemedicine Within Opioid Treatment Programs: Qualitative Study
Source: J Med Internet Res. 2024 Jun 12;26:e53049. doi: 10.2196/53049 (PMC11208831; doi:10.2196/53049)
Supplement: Multimedia Appendix 1 [file jmir_v26i1e53049_app1.pdf]

Multimedia Appendix 2. COREQ Checklist

Dickerson SS, George SJ, Ventuneac A, et al. Care Integration for Hepatitis C Virus Treatment through Facilitated Telemedicine within Opioid Treatment Programs: A Qualitative Study

The table below illustrates how and where (when applicable) our study addresses each item in the COREQ checklist.

**Characteristic**	**Guide questions/description**	**Manuscript page number**	**Description or relevant text from manuscript.**
**Domain 1: Research team and reflexivity**			
Personal Characteristics			
1. Interviewer/facilitator:	Which author/s conducted the interview or focus group?	Manuscript, page 3	"SSD interviewed all participants from January-July 2021."
2. Credentials	What were the researcher's credentials? E.g. PhD, MD		Ph.D.
3. Occupation	What was their occupation at the time of the study?		Dr. Dickerson is Professor of Nursing, Chair of Faculty Development and one of the senior scholars and co-founders of Hermeneutic Phenomenology Institute.
4. Gender	Was the researcher male or female?		Dr. Dickerson is female.
5. Experience and training	What experience or training did the researcher have?	Manuscript, pages 3	Suzanne S. Dickerson, PhD[1],<br>Saliyah J. George, MPH[2],<br>Ana Ventuneac, PhD[3],<br>Arpan Dharia, MD[4],<br>Andrew H. Talal, MD, MPH[4]<br><br>[1]Division of Tenured and Tenure-track Faculty Development, School of Nursing, University at Buffalo, Buffalo, NY, USA.<br>[2]Division of Liver Diseases, Department of Medicine, Icahn School of Medicine at Mount Sinai, New York,<br>[3]START Treatment & Recovery Centers, Brooklyn, NY<br>[4]Division of Gastroenterology, Hepatology, and Nutrition, Department of Medicine, University at Buffalo, Buffalo, NY, USA<br>Dr. Dickerson is an expert in qualitative research and has been a member of the Advanced Hermeneutical Institute for the past three decades. She has recently been a co-author on a definitive book on the topic entitled "Doing Hermeneutic Phenomenological

			Research: A practical guide" (2020, Sage Publication Inc., ISBN 978-1-5264-8573-1).<br><br>"The multidisciplinary analysis team included a hermeneutic phenomenology expert (SSD), a social psychologist (AV), and a case manager (SJG) who performed the initial thematic coding. Subsequently, the study principal investigator (AHT) and study director (AD) joined the analysis team for the final coalescence of themes."
Relationship with participants			
6. Relationship established	Was a relationship established prior to study commencement?		Yes, all interviewed participants were staff who participated in a study sponsored by the Patient-Centered Outcomes Research Institute (PCORI) comparing hepatitis C virus (HCV) treatment through facilitated telemedicine integrated into opioid treatment programs (OTPs) to offsite referral. The study was conducted in 12 OTPs throughout New York State. All interviewed staff participated in the facilitated telemedicine encounters (i.e., clinical staff), supported patients undergoing HCV treatment through telemedicine (i.e., patient engagement staff), or were responsible for HCV medication dispensing or side effect management (i.e., nursing staff). We also interviewed OTP administrators who were responsible for study planning, implementation, and conduct at their sites.
7. Participant knowledge of the interviewer	What did the participants know about the researcher? e.g. personal goals, reasons for doing the research-		Yes, all interviewed participants were aware of the intent of the research from their involvement in the randomized clinical trial that was initiated in 2017. During the conduct of the randomized trial, we conducted a variety of activities to increase site engagement including annual learning and staff appreciation lunches at each site that fostered awareness of the trial among all site staff. We also actively educated all site staff about the basics of HCV.

8. Interviewer characteristics	What characteristics were reported about the interviewer/ facilitator? e.g. bias, assumptions, reasons and interests in the research topic		Dr. Dickerson explained that she is a Professor of Nursing and that she has been working with the study team for several years. She explained that her initial experience with the study team commenced in 2017 when she participated in a thematic analysis of people with opioid use disorder testimonials of hepatitis C virus (HCV) treatment through telemedicine integrated into OTPs. Since that time, Dr. Dickerson has been a co-author on six additional publications including the current manuscript.
**Domain 2: Study design**			
Theoretical framework			
9. Methodological orientation and Theory	What methodological orientation was stated to underpin the study? e.g. grounded theory, discourse analysis, ethnography, phenomenology, content analysis	Manuscript, page 1	"We used hermeneutic phenomenology to reveal understandings of human situations as experienced within a context of time, place, and situational influences. In this approach, we gain an understanding of the experience of a technology from a perspective of health care as a human experience interacting with one another versus a replacement of social aspects of care. In a post positivist/interpretive framework, hermeneutic phenomenology uses an 'a priori' (i.e., no preconceived theory) approach to formulate open-ended questions, interpret the interview text, and explicate common meanings and shared practices. Themes are revealed through interpretation of the language of experiences, as portrayed in staff narrative interviews. For details concerning hermeneutic phenomenology, please see and Multimedia Appendices 1 and 2."
Participant selection			
10. Sampling	How were participants selected? e.g. purposive, convenience, consecutive, snowball	Manuscript page 2	"We utilized purposive sampling to recruit OTP staff and administrators involved in HCV care integration at least one year after initiation of facilitated telemedicine. We interviewed approximately four participants per site representing 11 sites. The study was approved by each sites' institutional

			review board. We interviewed 45 participants including 16 clinical, 15 administrative, and 14 support staff (Table 1)."
11. Method of approach	How were participants approached? e.g. face-to-face, telephone, mail, email	Appendix 1, page 3	"Participant recruitment<br>Initially, study-supported case managers categorized each OTP's staff roster based upon extent of facilitated telemedicine involvement (Appendix Table). We invited those with at least moderate involvement in the RCT by email or verbal communication, with a limit of two reminders for non-responses."
12. Sample size	How many participants were in the study?	Manuscript, page 2	**"**We interviewed 45 participants including 16 clinical staff, 14 support staff, and 15 administrative staff (Table 1)."
13. Non-participation	How many people refused to participate or dropped out? Reasons?	Manuscript, page 3	"No participants prematurely discontinued the interview."
Setting			
14. Setting of data collection	Where was the data collected? e.g. home, clinic, workplace	Manuscript, page 3	**"**Interviews were recorded by Zoom, transcribed, de-identified and verified by staff for accuracy. One participant chose to only have audio recorded. The interviews lasted 30 to 60 minutes."
15. Presence of non-participants	Was anyone else present besides the participants and researchers?	N/A	Only the participants and the researcher were present at the time of the interview.
16. Description of sample	What are the important characteristics of the sample? e.g. demographic data, date.	Table 1	Demographic data from the study participants are included in Table 1.
Data collection			
17. Interview guide	Were questions, prompts, guides provided by the authors? Was it pilot tested?	Manuscript, page 3	"We developed separate interview guides for staff and administrators (supplemental material). The initial open-ended question inquired about what the participants viewed as the most important aspect of integrated care through telemedicine. If not discussed in the open-ended inquiry, we

		Appendix 1	subsequently utilized probes for further elaboration on participants' experiences of facilitated telemedicine and integration frameworks.<br><br>Interview guides (pages 7-10) and a description of their development (page 3) are included in supplementary material.
18. Repeat interviews	Were repeat interviews carried out? If yes, how many?	N/A	No repeat interviews were performed.
19. Audio/visual recording	Did the research use audio or visual recording to collect the data?	Manuscript, page 3	"Interviews were recorded by Zoom, transcribed, de-identified and verified by staff for accuracy. One participant chose to only have audio recorded."
20. Field notes	Were field notes made during and/or after the interview or focus group?	Manuscript, page 3	"The analysis team interpreted the transcripts and field notes in a reflective process that followed iterative steps."
21. Duration	What was the duration of the interviews or focus group?	Manuscript, page 3	"The interviews lasted 30 to 60 minutes."
22. Data saturation	Was data saturation discussed?	Appendix 1, page 4	"We conducted interviews until the team determined that the interpretations were comprehensive and warranted. "
23. Transcripts returned	Were transcripts returned to participants for comment and/or correction?	Manuscript, page 3	"Interviews were recorded by Zoom, transcribed, de-identified and verified by staff by comparing transcriptions against the recording for accuracy… The interviewer confirmed the final transcript version prior to analysis."
**Domain 3: analysis and findings**			
Data analysis			
24. Number of data coders	How many data coders coded the data?	Manuscript, page 3	"The multidisciplinary analysis team included an HP expert (SSD), a social psychologist (AV), and a case manager (SJG), who performed the initial thematic coding. Subsequently, the study principal investigator (AHT) and study director (AD) joined the analysis

			team for the final coalescence of themes.”
25. Description of the coding tree	Did authors provide a description of the coding tree?	Manuscript, page 3<br><br>Appendix 1, page 3	“The analysis team interpreted the transcripts and field notes in a reflective process that followed iterative steps. The team met weekly to identify emergent themes with supportive quotes, which were discussed and refined in an iterative fashion.”<br>“The research team began the analysis with the initial interview transcript that each team member analyzed. Each member initially provided a separate interpretive summary, including preliminary themes with initial quotes, that were shared in weekly team meetings. Team members reviewed each subsequent interview transcript for overall understanding and continued to add to the interpretive summaries identifying preliminary themes with supporting quotes. These summaries were shared in the weekly team meetings, and team members returned to previous interpretations for comparisons.”
26. Derivation of themes	Were themes identified in advance or derived from the data?	Manuscript, page 3<br><br>Appendix 1, page 3-4	“The team met weekly to identify emergent themes with supportive quotes, which were discussed and refined in an iterative fashion.”<br><br>“Ongoing team discussion and dialogue refined developing themes and interpretations, comparing transcripts across interviews for commonalities and differences.”
27. Software	What software, if applicable, was used to manage the data?	Appendix 1, page 4	“We used NVivo (QSR International) to manage quotes and related themes.”
28. Participant checking	Did participants provide feedback on the findings?	Appendix 1, page 4	While we did not provide an opportunity for interviewed participants to comment on the findings, <u>we have shared high-level preliminary results of the analysis with several stakeholders including several members of the study Sustainability Committee and the Data Safety and Monitoring Board</u>. Relevant stakeholders have included leadership

			at study sites (who were not interviewed) or at other methadone programs throughout New York State, members of a coalition of methadone programs in New York State, and relevant individuals from the New York State Department of Health. Additionally, the analysis team was comprised of individuals who had extensive experience working in OTPs, with the opioid use disorder patient population, or who were employed by OTPs. All five members of the analysis team are in full agreement with the findings of the work as described in the submitted manuscript.
	Reporting		
29. Quotations presented	Were participant quotations presented to illustrate the themes / findings? Was each quotation identified? e.g. participant number	Manuscript, pages 3-10, Table 2	Yes, quotations are presented and are identified as to whether they were derived from a staff member or an administrator to protect participant identity. Since many sites have only one person per job category (i.e., one administrator), we were concerned that even the use of a study number might not preclude identification of the interviewee. This concern is augmented since the names and locations of the sites participating in the randomized trial are publicly available. The lack of explicit indication of the speaker's location reduces the likelihood of identification.
30. Data and findings consistent	Was there consistency between the data presented and the findings?	Manuscript, pages 3-10	The interpretation was warranted, and exemplary themes provide evidence of the interpretation.
31. Clarity of major themes	Were major themes clearly presented in the findings?	Manuscript, pages 3-10	"Participant interviews revealed four themes related to integration of facilitated telemedicine into a behavioral setting (Figure 1)."
32. Clarity of minor themes	Is there a description of diverse cases or discussion of minor themes?	Manuscript, pages 3-10	Yes, minor themes are discussed when adding understanding to the main theme and are supported by quotations. There were no diverse cases.
