# Supplementary material for: Care Integration for Hepatitis C Virus Treatment Through Facilitated Telemedicine Within Opioid Treatment Programs: Qualitative Study
Source: J Med Internet Res. 2024 Jun 12;26:e53049. doi: 10.2196/53049 (PMC11208831; doi:10.2196/53049)
Supplement: Multimedia Appendix 2 [file jmir_v26i1e53049_app2.pdf]

Multimedia Appendix 1. Methodology Supplement and Interview Guides

Dickerson SS, George SJ, Ventuneac A, et al. Care Integration for Hepatitis C Virus Treatment through Facilitated Telemedicine within Opioid Treatment Programs: A Qualitative Study

Supplement 2:

	Page
1) Methodology supplement	2
2) Appendix Table	6
3) Interview guides	
a. Interview guide for opioid treatment program staff	7-8
b. Interview guide for opioid treatment program administrators	9-10

Methodology supplement

Hermeneutic phenomenological research

As outlined in the textbook Doing Hermeneutic Phenomenological Research: A Practical Guide [1], we used the hermeneutic phenomenological research approach, which uses language to understand the temporal meaning of being-in-the-world that is influenced by the philosophical underpinnings and stances of Martin Heidegger and Hans-Georg Gadamer. This philosophy guides the questioning process using an 'a priori' open-ended approach (i.e., without preconceived theory or framework) to interview participants on their day-to-day life based on the experiences of the phenomenon of HCV care integration of facilitated telemedicine into opioid treatment programs. These interviews are the basis of understanding of the past, present, and future possibilities that forms the horizon of understanding. Through the researchers' interpretations of the participants' language, the horizon of understanding fuses, informing taken-for-granted meanings. For Heidegger, the spoken language is the primary means of communication and thinking, which provides narrative data for analysis by the researchers. The goal of hermeneutic phenomenological analysis is to understand the meanings of experiences in the specific situational context, that is explicated and beneficial to the future transferability of the phenomenon to similar contexts. We chose the hermeneutic phenomenological research approach, which seeks temporal meaning and situational context, over grounded theory, which centers on creating a substantive theory underpinned by basic social processes. Similarly, we chose the hermeneutic phenomenological approach, as opposed to interpretative phenomenological analysis (IPA), which is a psychology-informed technique that emphasizes the construct of cognition [1-5].

Interview guide development

The interview guides were initially developed by team consisting of the Hermeneutic Phenomenology expert (SSD), the overall study principal investigator (AHT), a study director, who also was a New York State Certified Substance Abuse Counselor (CASAC), an infectious diseases physician, and a clinical psychologist. Preliminary versions of the interview guide were assessed by health care professionals and opioid treatment program (OTP) staff not involved in the randomized controlled trial (RCT).

Participant recruitment

Initially, study-supported case managers categorized each OTP's staff roster based upon extent of facilitated telemedicine involvement (Appendix Table). We invited those with at least moderate involvement in the RCT by email or verbal communication, with a limit of two reminders for non-responses.

Analytic Process

The research team began the analysis with the initial interview transcript that each team member analyzed. Each member initially provided a separate interpretive summary, including preliminary themes with initial quotes, that were shared in weekly team meetings. Team members reviewed each subsequent interview transcript for overall understanding and continued to add to the interpretive summaries identifying preliminary themes with supporting quotes. These summaries were shared in the weekly team meetings, and team members returned to previous interpretations for comparisons. Ongoing team discussion and dialogue refined developing themes and interpretations, comparing transcripts across interviews for

commonalities and differences. When discrepancies arose amongst team members, we returned to the transcript for clarification and consensus. As new themes emerged, coalescence of themes continued resulting in an interpretation of the participants' experiences with nuanced understandings explicated in related themes. We conducted interviews until the team determined that the interpretations were comprehensive and warranted. We chose verbatim excerpts to explicate staff experiences of care integration with facilitated telemedicine. We used NVivo (QSR International) to manage quotes and related themes.

Preliminary versions of the study results were verified with a wide range of stakeholders consisting of healthcare providers, OTP staff not involved with the RCT, government officials, and policy makers responsible for the provision of substance use treatment to people with opioid use disorder.

Maintaining Rigor

We sustained reflective and iterative rigor throughout the entire interpretive process [1,6]. We utilized DeWitt and Ploeg's hermeneutic phenomenological framework to address balanced integration, openness, concreteness, resonance, and actualization. We ensured balanced integration through each team member's identification of their pre-understandings to enhance self-awareness of biases[7]. During analysis, we encouraged team members to consider their pre-understandings and biases while co-constituting the participants' views and the researchers' interpretations. We attained openness by being reflexive of our own biases and using a systematic process of auditing the interpretive decisions realized through reflexive journaling. We achieved concreteness by providing context to situate the reader in the setting

that relates to everyday experiences in the opioid treatment program. Resonance is accomplished when the reader intuitively grasps the findings through the selected verbatim examples. Actualization was accomplished when the reader recognizes the future resonance (i.e., transferability) of these results to other similar situations.

Supplement references

1. Dibley L, Dickerson S, Duffy M, Vandermause R. Doing hermeneutic phenomenological research: a practical guide. London: SAGE; 2020. ISBN: 978-1-5264-8573-1.
2. Smith JA. Beyond the divide between cognition and discourse: Using interpretative phenomenological analysis in health psychology. Psychol Health 1996;11(2):261-71. [DOI:10.1080/08870449608400256][
3. Brocki JM, Wearden AJ. A critical evaluation of the use of interpretative phenomenological analysis (IPA) in health psychology. Psychol Health 2006;21(1):87-108. [DOI:10.1080/14768320500230185][
4. Smith JA. Reflecting on the development of interpretative phenomenological analysis and its contribution to qualitative research in psychology. Qual Res Psychol 2004;1:39-54.
5. Charmaz K. Constructing Grounded Theory. London: Sage; 2014. ISBN: 978-0-8570-2914-0
6. Ironside PM. Hermeneutics. In: Fitzpatrick JJ, Kazer MW, eds. Encyclopedia of nursing research 4th ed. New York: Springer Publishing Company; 2017. ISBN: 978-0-8261-3304-5
7. De Witt L, Ploeg J. Critical appraisal of rigour in interpretive phenomenological nursing research. J Adv Nurs 2006;55(2):215-229. [DOI:10.1111/j.1365-2648.2006.03898.x][Medline: 16866813]

Appendix Table: Criteria utilized to categorize staff by level of involvement in study

Staff category	Low involvement	Medium involvement	High involvement
Counselors	No study participant contact	Referral of at least one patient to study	At least one patient on prior or current case load,
Nurses	No study participant contact	Medication dispensing	Medication adherence, side effect identification, medication procurement
Clinical staff	No involvement with healthcare delivery to study participants.	Minimal involvement in healthcare delivery, such as attending telemedicine visits as substitute.	Active and sustained involvement with healthcare delivery to study participants.
Administration	No involvement in study planning, implementation, and conduct	Some involvement in study planning, implementation, and conduct	Active involvement in study planning, implementation, and conduct

Interview Guide – Staff

Opening Script

As you know, we are doing research to try to learn more about the integration of HCV care and opioid use disorder care, specifically via telemedicine. While I have a number of questions I am going to ask you, I am most interested in your own individual experiences and opinions of care administration and coordination, so if you have thoughts or experiences you would like to share with me, even if I have not asked about them, please feel free to tell me. You may refuse to answer any questions I ask you, and you may end the interview at any time for any reason.

Instructions for interviewer: please adhere to open-ended questions as below. In a few situations, follow-up questions are permissible as we are interested in obtaining specific information regarding certain domains.

Restatement of consent
Is it okay start the interview?
I will be turning on the recorder now/start recording now (if via teleconferencing platform, e.g., Zoom).

Interview Guide
Initial open-ended question

1) What experience stands out in your mind regarding the integration of telemedicine for hepatitis C treatment?

Workflow/internal/external regulation

2) What is a typical day like for you?
   a. What is your experience of the integration of telemedicine into the workflow of the OTP?
      Potential probes:
      - *What was your experience of support (or not) from study staff?*
      - *What was your experience of support (or not) from OTP colleagues?*
      - *What was your experience of support (or not) from your OTP administration?*
   b. What supporting activities would you recommend that could facilitate onsite healthcare delivery via telemedicine?
      Probes that emerged from the data collected:
      - ***Have you seen or been involved in the telemedicine encounter? What was it like?***
      - ***What do you think about the need to have eyes on the patients?***
      - ***Any suggested work arounds needed for telemedicine care?***

People and staff within the OTP and working environment

3) How would you describe the environment of your current OTP?
   Probes: (Instructions for interviewer: If participants provide examples of any of these explore more. If they do not, ask about these specifically).
   - *Level of trust between the clients and the staff?*
   - *Sense of community*
   - *Stigma*
   - *Compared to other medical settings*

Personalized care

4) Were there any issues with patients that were barriers to care?

   Potential probes:

   - *Can you give an example of your OTP support that facilitate (or not) delivering personalized care to clients?*
   - *What is your experience of telemedicine improving (or impairing) your ability to deliver personalized care?*

Measurement/Financial sustainability/Future growth

5) What about the future of telemedicine?

   Potential probes:

   - *What would you say are the areas for future growth for telemedicine and care integration?*
   - *What improvements in telemedicine delivery would you recommend to make the practice sustainable?*

6) What were important lessons learned about telemedicine situated in an OTP?

Additional

7) Is there anything else you would like to tell us about your experiences that we have not discussed?

Interview Guide - Administrators

Opening Script

As you know, we are doing research to try to learn more about the integration of HCV care and opioid use disorder care, specifically via telemedicine. While I have a number of questions that I am going to ask you, I am most interested in your own individual experiences and opinions as an OTP administrator, so if you have thoughts or experiences you would like to share with me, even if I have not asked about them, please feel free to tell me. You may refuse to answer any questions I ask you, and you may end the interview at any time for any reason.

Instructions for interviewer: please adhere to open-ended questions as below. In a few situations, follow-up questions are permissible as we are interested in obtaining specific information regarding certain domains.

Restatement of consent
Is it okay start the interview?
I will be turning on the recorder now/start recording now (if via teleconferencing platform, e.g., Zoom).

Interview Guide
Initial open-ended question
1) What experience stands out in your mind regarding the integration of telemedicine for hepatitis C treatment?

Workflow/internal/external regulation
2) Could you describe your experiences with onsite telemedicine in your setting related to the following domains?
    - Workflow and staffing
    - Internal regulations
    - External regulations
3) What activities would you recommend to support onsite telemedicine?

Measurement/Financial sustainability
4) Can you give examples of specific data needs you have that would be helpful in supporting telemedicine?
    Probes: (Instructions for interviewer: If participants provide examples of any of these, explore more: quality of care related to HCV treatment via telemedicine, HCV medication adherence, time required to conduct telemedicine visits, and patient satisfaction).
5) Can you give examples of how reimbursement affects the sustainability of onsite telemedicine?
6) Can you give examples of staffing needs to conduct onsite telemedicine?

Treatment integration What other uses for integrated care in your setting?
7) Can you give us examples of how your organization has integrated medical care and other services into its practice?
8) Can you give examples how telemedicine could be used for integration of medical and behavioral treatment in your organization?

People/staffing

9) How would you describe the environment of your current OTP?

Probes: (Instructions for interviewer: If participants provide examples of any of these explore more. If they do not, ask about these specifically).

- *Level of trust between the clients and the staff?*
- *Sense of community*
- *Stigma*
- *Compared to other medical settings*

Future growth

10) What about the future of telemedicine?

Probe:

- *What would make onsite telemedicine in your OTP (for HCV as well as other medical and mental health problems) an intervention that you would adopt long term?*

11) What were important lessons learned about telemedicine situated in an OTP?

Additional

12) Is there anything else you would like to tell us about your experiences that we have not discussed?
